# Time-Dependent Stabilization of Hypoxia Inducible Factor-1α by Different Intracellular Sources of Reactive Oxygen Species

**DOI:** 10.1371/journal.pone.0038388

**Published:** 2012-10-29

**Authors:** Maura Calvani, Giuseppina Comito, Elisa Giannoni, Paola Chiarugi

**Affiliations:** 1 Department of Biochemical Sciences, University of Florence, Florence, Italy; 2 Istituto Toscano Tumouri and “Center for Research, Transfer and High Education DenoTHE”, Florence, Italy; University of Tennessee, United States of America

## Abstract

Intratumoral hypoxia is a major obstacle in the development of effective cancer chemotherapy, decreasing the efficacy of anti-neoplastic drugs in several solid tumours. The hypoxic environment, through its master regulator hypoxia inducible factor-1 (HIF-1), is able to maintain an anti-apoptotic potential through activation of critical genes associated with drug resistance. Besides affecting metabolism and motility of tumour cells, hypoxia also paradoxically increases production of reactive oxygen species (ROS), which contribute to stabilize HIF-1 through a redox-mediated inhibition of its proteolysis. Here we reported that 1% O_2_ hypoxia increases the resistance of human metastatic melanoma cells to conventional chemotherapy with etoposide, and that the increase in chemoresistance strongly depends on ROS delivery due to hypoxia. We reported a biphasic redox-dependent role of HIF-1, involving mitochondrial complex III and NADPH oxidase as oxidants sources, synergising in enhancing survival to chemotherapy. The feed-forward loop engaged by hypoxia involves first an HIF-1-dependent vascular endothelial growth factor-A (VEGF-A) autocrine production and, in the later phase, activation of NADPH oxidase from VEGF/VEGFR2 interaction, finally leading to a further redox-dependent long lasting stabilization of HIF-1. We therefore identified a redox-dependent circuitry linking hypoxia-driven ROS to VEGF-A secretion and to enhanced melanoma cell survival to etoposide chemotherapy.

## Introduction

Melanoma is the most aggressive form of skin cancer and its advanced stages are inevitably associated with a poor prognosis, due to their resistance to conventional therapeutic agents. In particular, the resistance to undergo apoptosis in response to chemotherapy and other environmental cues gives rise in aggressive melanoma to a selective advantage for tumour progression, metastasis formation as well as for resistance to therapy [1], .

Acquired resistance to chemotherapy is generally considered to be the result of the gradual selection of mutant subpopulations, genetic mutations and biochemical alterations. Of note, tumour microenvironment is known to contribute in different ways to drug resistance essentially through increasing cancer mutation rate or creating a selective pressure favouring resistant and aggressive populations [3]. Two interesting components of the tumour microenvironment are hypoxia and reactive oxygen species (ROS), often reported as strong activators of cancer progression and correlated with poor outcome for patients [4], [5].

Hypoxia is frequent in solid tumours, being the natural consequence of the increased oxygen diffusion distance due to tumour expansion [6]. The transcriptional response of mammalian cells to hypoxia is largely mediated by hypoxia-inducible factor-1 (HIF-1) [7]–[9]. HIF-1 is a basic helix-loop-helix transcription factor composed of an HIF-1β subunit, which is constitutively expressed, and an HIF-α subunit, which is strongly up-regulated under hypoxic conditions. At least 3 isoforms of the α subunit have been identified so far, although HIF-1α is the master regulator of the transcriptional response to hypoxia. In normoxic conditions, HIF-1α is degraded by a mechanism involving hydroxylation of 2 prolyl residues, ubiquitination and proteasomal degradation through a VHL-dependent pathway. Stabilization of HIF-1α is also influenced by genetic alterations, as well as by growth factors, hormones and cytokines produced by both tumour and stromal cells [10]. Under hypoxic condition HIF-1 coordinates the expression of many genes that orchestrate angiogenesis and cancer cell metabolism reprogramming, including GLUT1 and GLUT3, glycolytic enzymes, vascular endothelial growth factor (VEGF), erythropoietin (EPO), heme oxygenase-1 (HO-1), etc [11].

Under hypoxic conditions, the hydroxylation of HIF-1α is inhibited, and HIF-1α is stabilized and competent to activate transcription of target genes. ROS, in turn, inactivate prolyl hydroxylases (PHDs) through oxidation of the ferrous ion that is essential for their catalytic mechanism, and hence stabilize HIF-1α. Vitamin C has been shown to decrease HIF-1 levels by preventing the oxidation of the catalytic ferrous ion [12]–[14]. In keeping, it has been recently reported that the anti-tumorigenic effect of antioxidants as N-acetyl cysteine (NAC) and vitamin C in murine models of Myc-mediated tumorigenesis are indeed HIF-1-dependent [15].

Hypoxia is closely related to oxidative stress. Of note, the genetic disruption of the PHD1 gene in hypoxic mice lowers oxygen consumption in the mitochondria of skeletal muscle, reduces oxidative stress, and eventually enhances cellular survival [16]. In keeping, we have recently observed that during hypoxia melanoma cells are subjected to persistent oxidative stress due to increase their intracellular concentration of ROS, due to mitochondrial complex III deregulation [17]. Mitochondrial ROS have been largely involved in ROS development and consequent HIF-1 stabilization under hypoxia [18], as well as for non hypoxic conditions [19]. Beside mitochondria, NADPH oxidases have been greatly involved in ROS production and in redox-dependent HIF-1 stabilization, although mainly in normoxic conditions. Indeed, NADPH-driven ROS have been involved in HIF-1 activation upon Src activation, after hyperthermia treatments, or in macrophages activated by lipopolysaccharides (LPS) [20]–[22]. Of note, intermittent hypoxia induces a redox-dependent stabilization of HIF-1 in rat pheochromocytoma PC12 cells, which is dependent on NADPH oxidase-driven ROS, as well as Ca^2+^ and mTOR signalling. In any case, increasing ROS generation under hypoxia may trigger a redox adaptation response that enables cancer cells to survive, owing to increased tolerance of exogenous stress, up-regulation of survival molecules and increased ability for drug inactivation.

In this work we analysed the biological events, triggered by hypoxia, involved in melanoma resistance to conventional chemotherapy. Using HS29-4T human metastatic melanoma cells treated with etoposide, we proved that hypoxia-driven ROS orchestrate a biphasic redox-dependent cellular response that leads cells to escape apoptosis induced by both etoposide and hypoxic hostile environment. We found that mitochondrial ROS (mtROS) and NADPH oxidase ROS (noxROS) cooperate in HIF-1 stabilization, although with a different time course. We identified a redox-dependent circuitry linking hypoxia-driven ROS to VEGF secretion and to increased survival and chemoresistance.

## Results

### Hypoxia protects Hs29-4T cells from etoposide induced apoptosis

**Figure 1:**
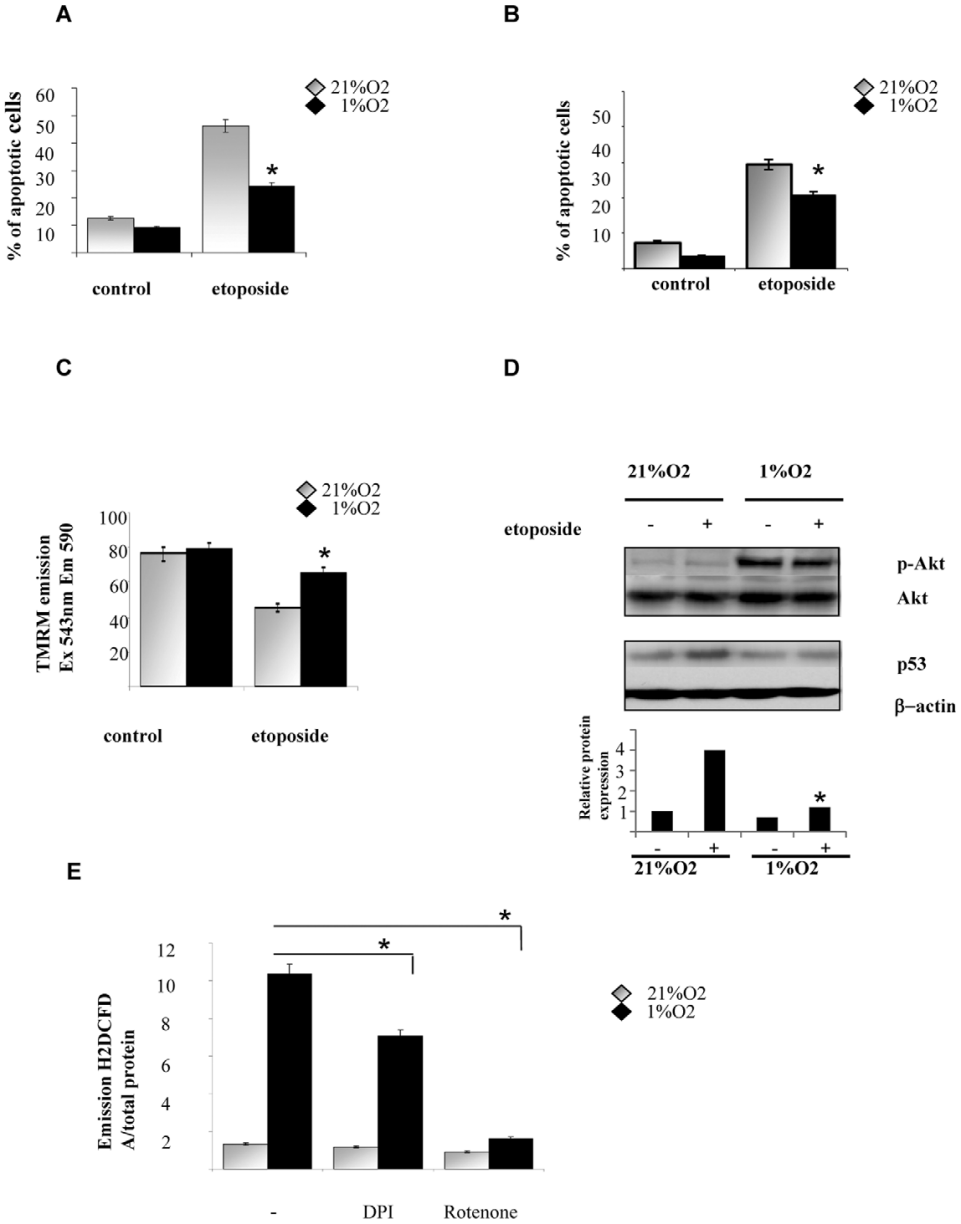
Hypoxia induces cell survival in melanoma cells. Hs29-4T cells were serum-starved for 24 h and then incubated under normoxia or hypoxia in the absence or presence of etoposide (50 µM) for additional 24 h. Different parameter revealing cell death or cell survival were examined. (**A**) Percentage of apoptotic cells was measured with Annexin V staining *versus* propidium iodide-positive cells; averages SD values are shown in the bar graph. *P<0,001 versus etoposide-treated under hypoxia. (**B**) Cells were stained with specific antibody against caspase 3. Cells positive to caspase 3 staining were revealed by flow cytometric analysis; averages ±SD values are shown in the bar graph. *P<0,001 versus etoposide-treated under hypoxia. (**C**) Living cells were stained with TMRN and mitochondrial fluorescence (excitation 543 nm, emission 590 nm) was quantified by fluorimetric assay (FluoroSkan) and normalized on protein content; averages ±SD values are shown in the bar graph. *P<0,001 versus etoposide-treated under hypoxia. (**D**) After cells lysis p-Akt and p-53 proteins accumulation were assessed with western blotting. β-Actin is shown as loading control. Image quantification reports fold increase in protein expression. (**E**) Hs29-4T cells were serum-starved for 24 h, then incubated under normoxia or hypoxia for 24 h in the absence or presence of specific inhibitors of the various form of ROS, namely DPI (5 µM) and rotenone (1 µM). Hydrogen peroxide production was evaluated by DCDF-DA and normalized on protein content. *P<0,001 versus untreated control under hypoxia.

In order to investigate how chemoresistance develops in melanoma, we choose human metastatic melanoma cell line (Hs29-4T) treated with etoposide under hypoxic conditions. Our results indicate that the number of apoptotic cells is greatly reduced in hypoxic etoposide stimulated cells as revealed by the 50% decrease of positivity to Annexin V end Caspase 3 staining (Fig. 1A–B). Cells viability was demonstrated by TMRM staining. In particular, Fig. 1C shows a consistent increase of fluorescence in cells treated with etoposide in hypoxia if compared with normoxic control, in keeping with a more resistant and malignant phenotype. Moreover hypoxia induces phosphorylation of Akt protein in cells, independently from etoposide treatment (Fig. 1D). To confirm hypoxic induced survival we investigated if oxygen decrease affects p53 stabilization during in etoposide treatment. Etoposide added to HS29-4T in normoxia increases p53 protein phosphorylation leading cells to apoptosis; on the contrary p53 phosphorylation is not affected by etoposide when cells are exposed to hypoxia (Fig. 1D).

### Hypoxic-mediated oxidative stress induces survival in Hs29-4T cells

**Figure 2:**
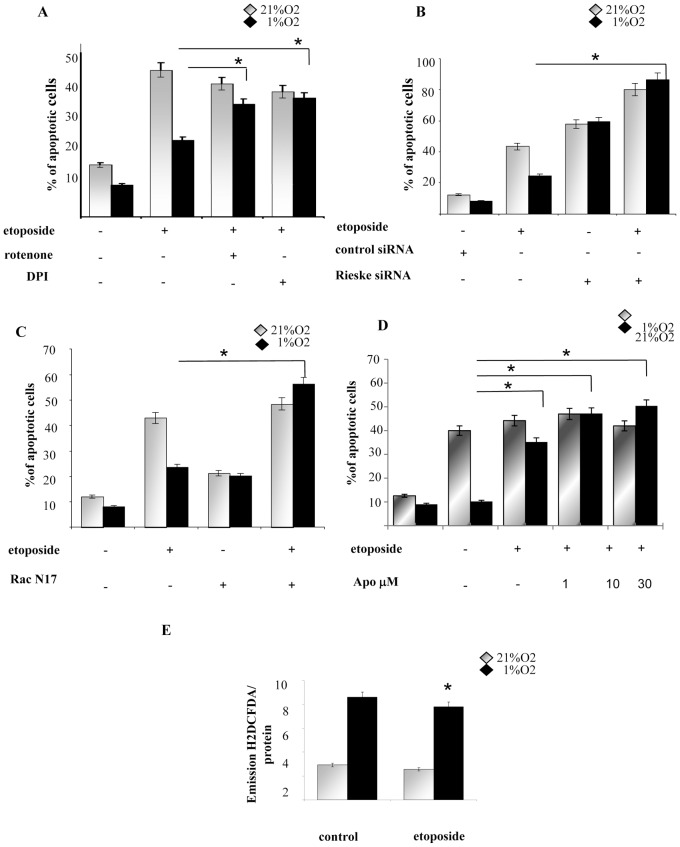
ROS induce survival in hypoxic melanoma cells. (**A**) Hs29-4T cells were serum-starved for 24 h, then incubated under normoxia or hypoxia for 24 h in the absence or presence of specific inhibitors of the various form of ROS, namely DPI (5 µM) and rotenone (1 µM). Percentage of apoptotic cells was measured with Annexin V staining *versus* propidium iodide-positive cells; *P<0,001 versus Etoposide treated control under hypoxia. (**B**) Cells were transfected with siRNA targeting Rieske Fe-S protein, serum-starved for 24 h and incubated in hypoxia and normoxia for additional 24 h. Percentage of apoptotic cells was measured by flow cytometry analysis of Annexin V *versus* propidium iodide-positive cells. *P<0,001 versus Etoposide treated control under hypoxia. (**C**) Cells were transfected with dominant negative RacN17 and after 24 h of serum starvation cells were exposed to 24 h of normoxia and hypoxia. Percentage of apoptotic cells was measured as in B. *P<0,001 versus Etoposide treated control under hypoxia. (**D**) Hs29-4T cells were serum-starved for 24 h and then incubated under normoxia or hypoxia in the absence or presence of etoposide (50 µM) for additional 24 h. Hydrogen peroxide production was evaluated by DCDF-DA and normalized on protein content. *P<0,001 versus Etoposide treated control under hypoxia. (**E**) Hs29-4T cells were serum-starved for 24 h, then incubated under normoxia or hypoxia for 24 h in the presence of Apocynin in dose crescent stimulation, 1, 10 and 30 µM. Percentage of apoptotic cells was measured with Annexin V staining *versus* propidium iodide-positive cells; *P<0,001 versus Etoposide treated control under hypoxia.

Firstly, to clarify the role of redox hypoxic signalling in melanoma resistance, we measured level of ROS during hypoxia. Cells exposed to hypoxia increase by 5-fold their level of ROS, if compared with normoxic control. The antioxidants rotenone and DPI, affecting mitochondrial and NADPH oxidase-driven ROS delivery, reduce the level of intracellular ROS during hypoxia (Fig. 1E), suggesting that both these sources of ROS (mtROS and noxROS) are active in hypoxic stimulated Hs29-4T cells. We then evaluated how ROS affect cells survival. The results indicate that hypoxia markedly inhibits etoposide-induced apoptosis and both DPI and rotenone revert the hypoxic protective effect, suggesting that mtROS and noxROS are key mediators also for melanoma survival during hypoxia (Fig. 2A). To validate our data, we selectively inhibited mtROS generation by siRNA targeting Rieske Fe-S protein (component of Ubiquinol-cytochrome-c reductase in the mithocondria) or using the dominant negative small GTP-ase Rac-1 (RacN17), a key upstream regulator of NADPH oxidase. The inhibition of ROS generations by these approaches increases the number of hypoxic apoptotic cells, either treated or not with etoposide (respectively about 6 fold and 3,5 fold, as indicated in Fig. 2B, C). Apocinyn, a selective inhibitor of NADPH oxidase, reverts hypoxic induced survival in etoposide-stimulated hypoxic cells in a dose-dependent manner (Fig. 2D). Many anticancer drugs act, at least partially, through intracellular delivery of toxic ROS and an increased antioxidant capacity is a frequent escape strategy for cancer cells under anti-tumour treatment. Nevertheless, we observed that etoposide does not affect ROS levels, independently from exposure to hypoxia, thereby excluding a direct involvement of the drug in ROS generation (Fig. 2E).

### HIF-1α mediates cells survival in melanoma cells Hs29-4T

**Figure 3:**
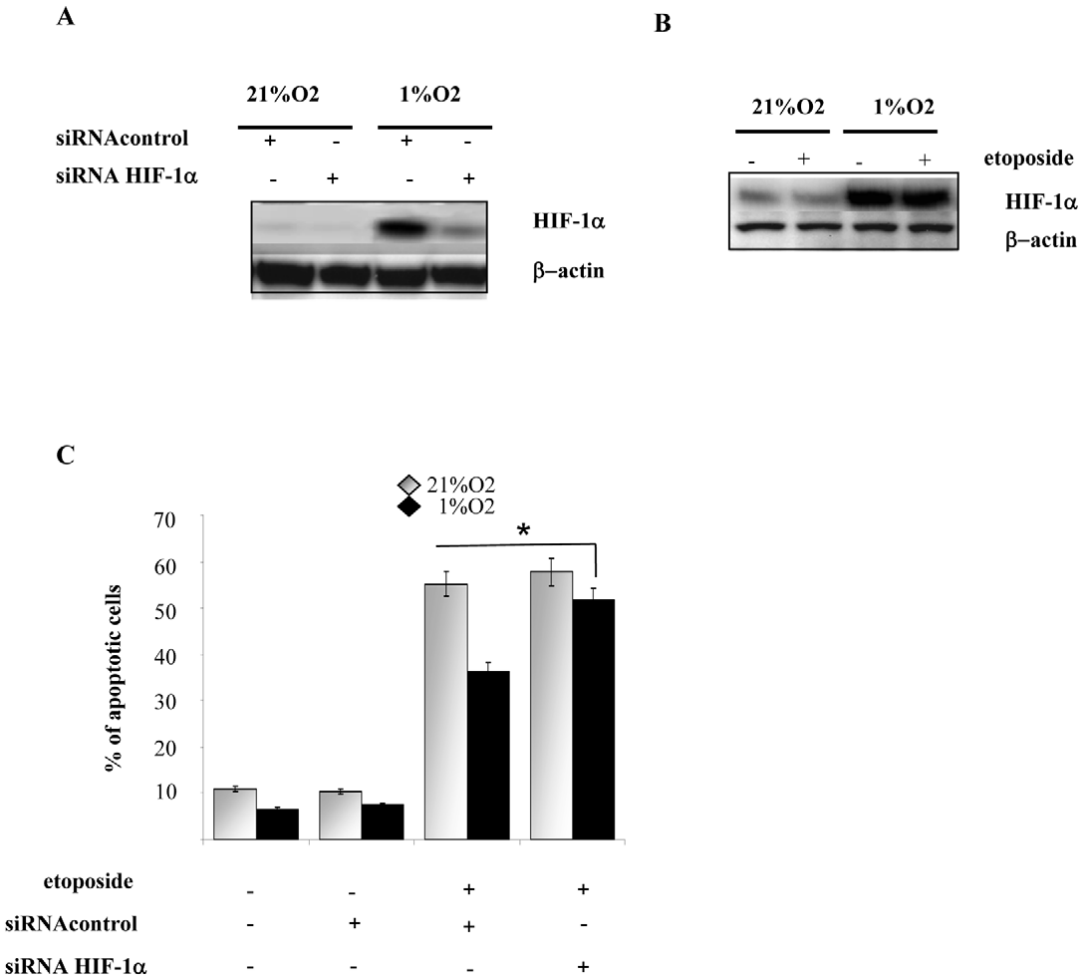
HIF-1α leads melanoma cells survival. (**A**) Cells were transfected with siRNA targeting HIF-1α and with a control siRNA. After 24 h of serum starvation, cells were exposed for 24 h to normoxia or hypoxia. Percentage of apoptotic cells was measured by flow cytometry analysis of Annexin V *versus* propidium iodide-positive cells. *P<0,001 versus Etoposide treated control under hypoxia. (**B**) Hs29-4T were transfected with siRNA targeting HIF-1α and with a control siRNA. Cells were serum-starved for 24 h and then incubated under normoxia or hypoxia for additional 24 h. HIF-1α expression was analysed by immunoblot. β-Actin is shown as loading control. (**C**) Melanoma cells were serum-starved for 24 h and then were treated in the absence or presence of etoposide (50 µM) and incubated under normoxia or hypoxia for additional 24 h. HIF-1α expression was analysed by immunoblot. β-Actin is shown as loading control.

As HIFs transcription factors are implicated in tumour progression, metastasis and resistance to therapy, we investigated whether HIF-1 is involved in hypoxic-induced survival in melanoma. Cells were transiently transfected with siRNAs oligonucleotides targeting HIF-1α and, afterwards, cultured under hypoxia. First, we confirmed that silencing of HIF-1α is able to revert the ability of 1% hypoxia to elicit activation of the transcription factor (Fig. 3A). Second, we confirm that etoposide treatment does not further affect HIF-1 accumulation, autonomously with respect to oxygen concentration (Fig. 3B). Finally, the analysis of apoptotic cells during etoposide and hypoxia treatments, revealed that HIF-1α silencing under hypoxic conditions makes cells sensitive to therapy with etoposide, thereby reverting the protective effect exerted by hypoxia, by increasing the number of apoptotic cells by 1,5 fold (Fig. 3C).

### Hypoxia-induced ROS affect HIF-1α stabilization

**Figure 4:**
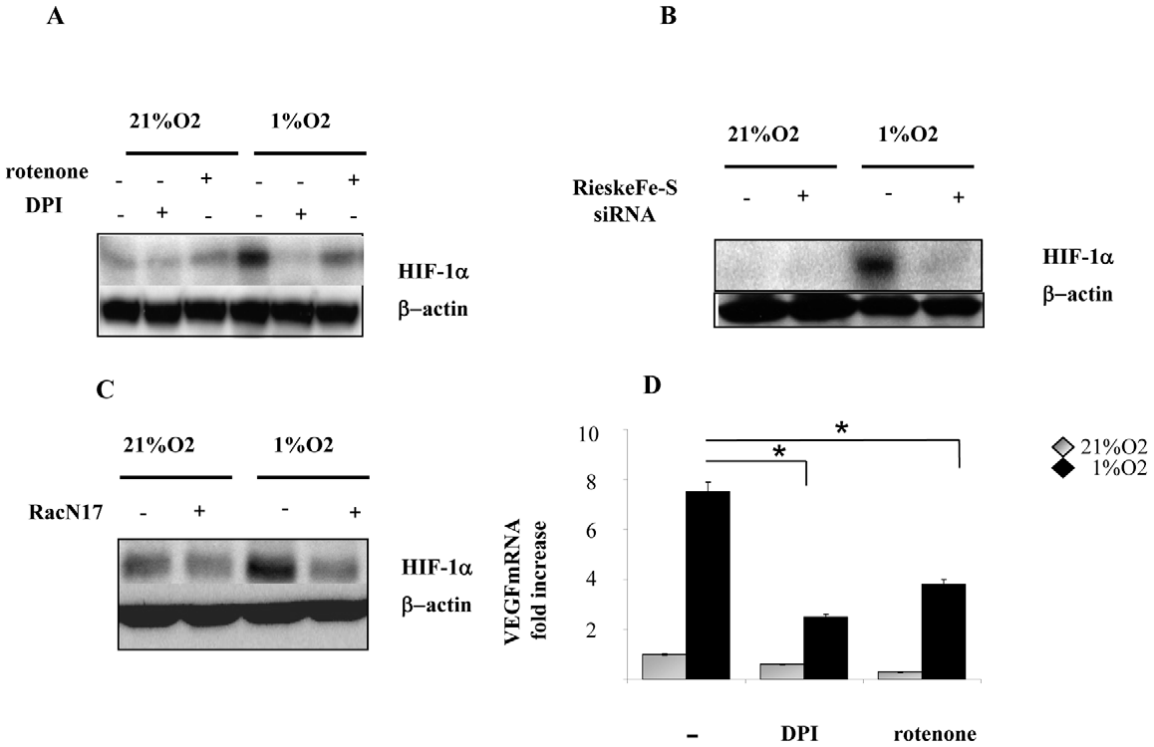
ROS stabilize HIF-1α protein under hypoxia. (**A**) Hs29-4T cells serum-starved for 24 h and incubated under normoxia or hypoxia for additional 24 h, in the presence or absence of DPI (5 µM) and rotenone (1 µM). HIF1-α expression was analysed with immunoblot. β-Actin is shown as loading control. (**B**) Cells were transfected with siRNA targeting Rieske Fe-S protein and after 24 h of serum starvation were incubated under normoxia and hypoxia for 24 h. HIF-1α expression was evaluated by immunoblot. β-Actin is shown as loading control. (**C**) Cells were trasfected with dominant negative RacN17, serum-starved for 24 h and incubated under hypoxic and normoxic conditions for additional 24 h. HIF-1α expression was analysed by immunoblot. β-Actin is shown as loading control. (**D**) Hs29-4T cells were serum-starved for 24 h and incubated under normoxia or hypoxia in the absence or presence of DPI (5 µM) and rotenone (1 µM). mRNA for VEGF was measured by real time PCR. *P<0,001 versus untreated control under hypoxia.

Previous results indicated clearly that both ROS and HIF-1 are mandatory for cells survival and that ROS are key molecules for HIF-1α stabilization. In order to understand the meaning of HIF redox regulation in cancer resistance, we analysed the effect of different antioxidants in HIF-1α accumulation.. Data show that both DPI and rotenone decrease HIF-1α protein expression, with DPI acting as a more effective inhibitor (Fig. 4A). The same results were achieved using Rieske Fe-S protein siRNA and dominant negative RacN17, thereby confirming a direct involvement of both mtROS and noxROS on HIF-1α expression during hypoxia (Fig. 4B,C). To verify if ROS affect HIF-1α transcriptional activity we measured VEGF-A mRNA, one of the main HIF-1α target genes and a key factor for *de novo* tumour angiogenesis. In hypoxic conditions, VEGF-A mRNA levels increase by 8-fold compared with normoxic control, while DPI and rotenone inhibit the hypoxic-induced increase of VEGF, thereby confirming a key role of ROS in regulating HIF-1-mediated function (Fig. 4D).

### VEGF-A mediates HIF-1α-dependent cell survival

**Figure 5:**
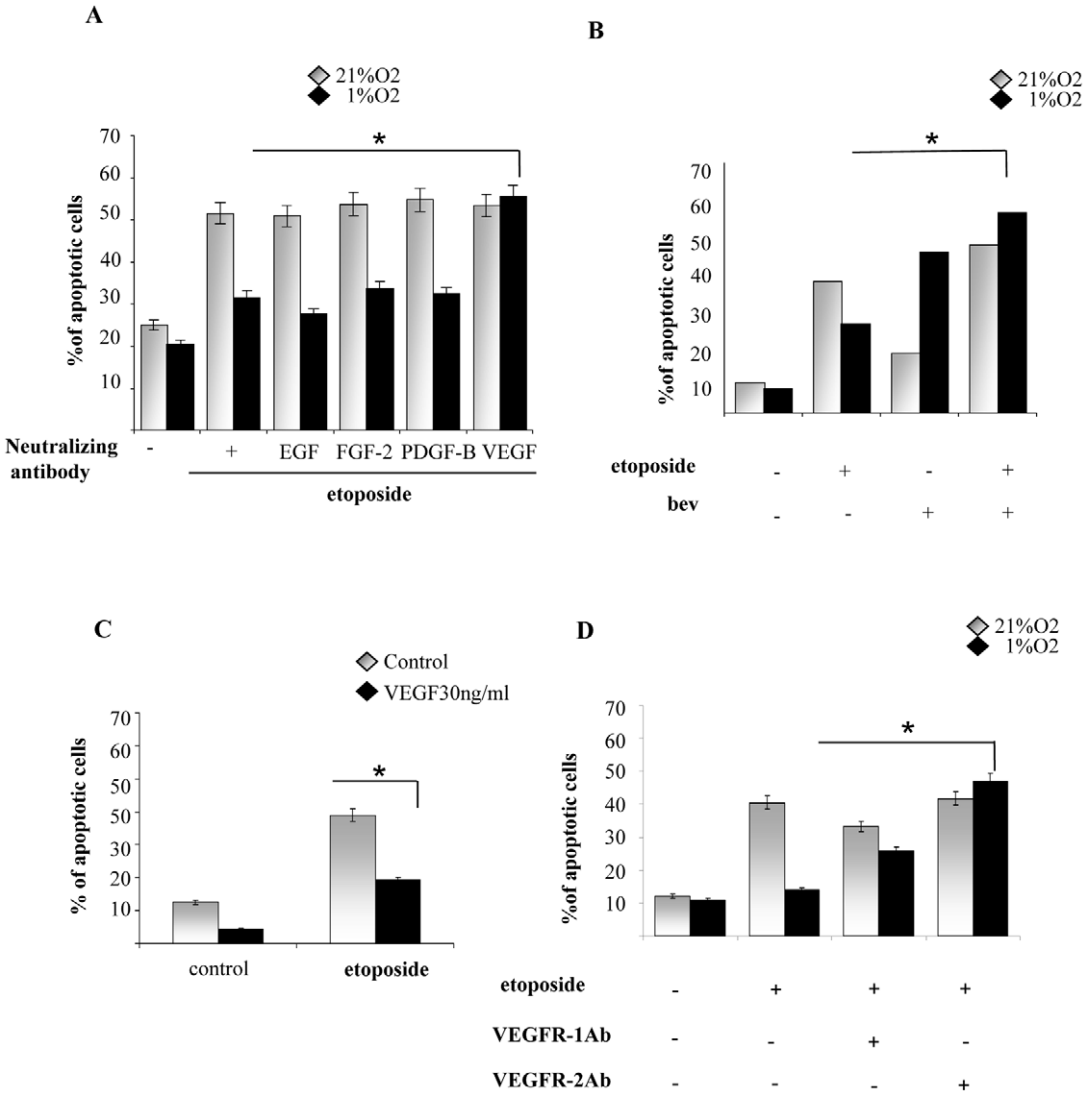
VEGF induces survival in hypoxic melanoma cells. HS29-4T cells serum-starved in for 24 h, then incubated under normoxia or hypoxia in the absence or presence of etoposide and treated with following conditions: (**A**) neutralizing antibody for 4 different growth factor, anti-EGF (50 ng/mL), anti-FGF-2 (1 µg/mL), anti-IGF-I (10 µg/mL), anti-PDGF-BB (100 ng/mL), and anti-VEGF (50 ng/mL) *P<0,001 versus untreated control under hypoxia. (**B**) VEGF (30 ng/ml); (**C**) bevacizumab 250 ng/ml; (**D**) neutralizing antibody for VEGF receptors KDR and Flt-1. Percentage of apoptotic cells was measured by Annexin V staining. *P<0,001 versus untreated control under hypoxia.

It is well known that many tumour cell lines produce large amounts of growth factors. Therefore we investigated whether autocrine production of growth factors are involved in survival to etoposide induced by hypoxia in melanoma cells. Cells were cultured in absence of exogenous growth factors, exposing them to either hypoxia and to neutralizing antibodies targeting growth factors that are frequently deregulated in human cancers, including EGF, FGF-2, IGF-I, PDGF-BB, and VEGF-A. Our results suggest that VEGF-A is the main responsible for the pro survival effect exerted by hypoxia, as its functional blockage with a specific neutralizing antibody increases the number of apoptotic cells of 40% (Fig. 5A). Bevacizumab (Avastin), a humanized anti-VEGF monoclonal antibody, commonly used in combination with chemotherapy for clinical treatment of patients, in our experimental system reverts completely the protection from cell death induced by hypoxia (Fig. 5B). Hs29-4T cells were than cultured under normoxic conditions in the presence or absence of VEGF-A. As shown in Fig. 5C, VEGF-A decreases the percentage of apoptotic cells compared to untreated control. VEGF mainly binds two tyrosine kinase receptors on endothelial cells: VEGF receptor-1 (Flt-1) and VEGF receptor-2 (KDR) [23]. VEGFRs are also expressed in non-endothelial cells, including haematopoietic stem cells and monocytes, as well as in a variety of tumour histotypes like prostate and colon cancers [24]. Our results indicate that addition of antibodies neutralizing both receptors, increases cells death in hypoxia. Nevertheless, the action of VEGF-R2 neutralizing antibody has a stronger effect (3-fold of increase) (Fig. 5D).

### VEGF autocrine loop is redox dependent

**Figure 6:**
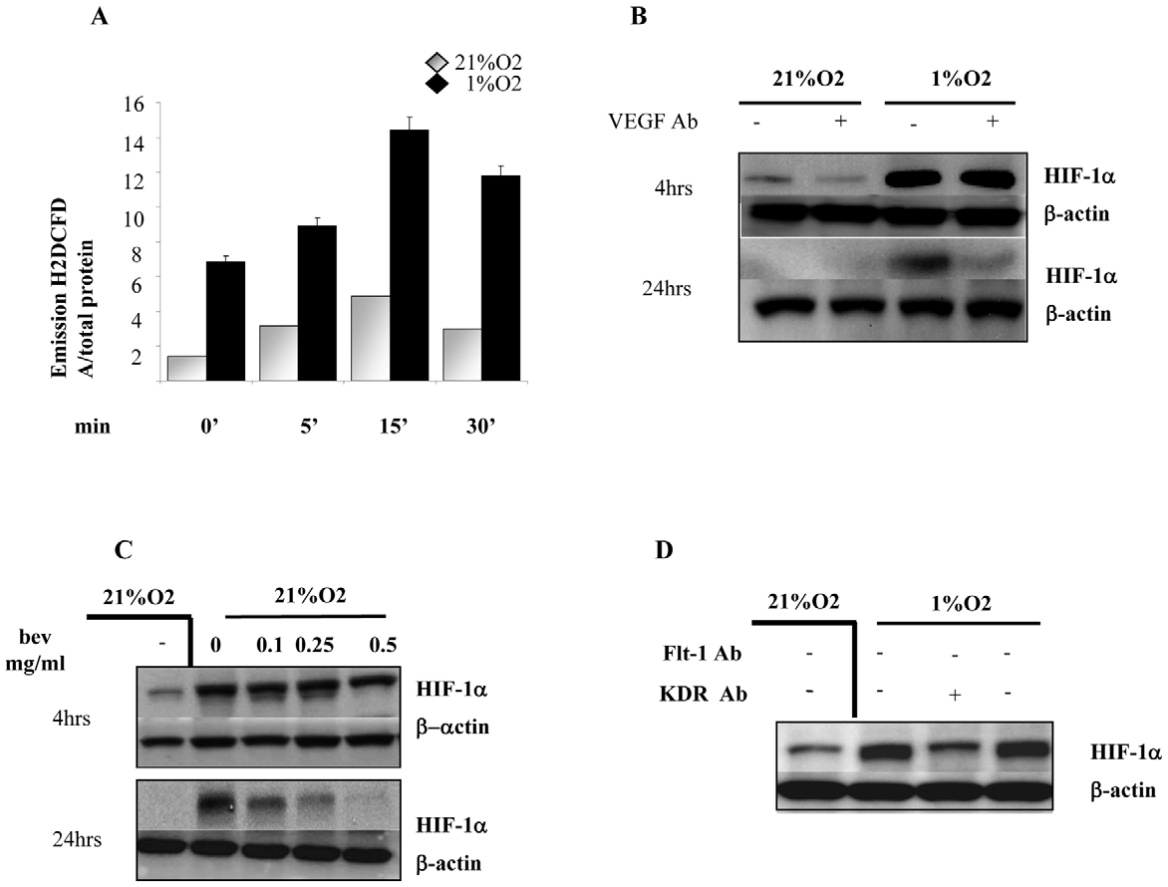
VEGF derived ROS stabilize HIF-1α protein. (**A**) Hs29-4T cells were serum-starved for 24 h and then incubated under normoxia or hypoxia in presence of recombinant VEGF 30 ng/ml from 0 to 30 min. Hydrogen peroxide production was evaluated with DCDF-DA and normalized on protein content. Cells were serum-starved for 24 h and incubated under normoxia or hypoxia for 24 h in the following different conditions: (**B**) neutralizing antibody for VEGF; (**C**) increasing concentrations of bevacizumab; (**D**) neutralizing antibody for KDR and Flt-1 receptors. HIF-1α expression was revealed by immunoblot. β-Actin is shown as loading control. *P<0,001 in A versus untreated control under hypoxia at 15 min.

**Figure 7:**
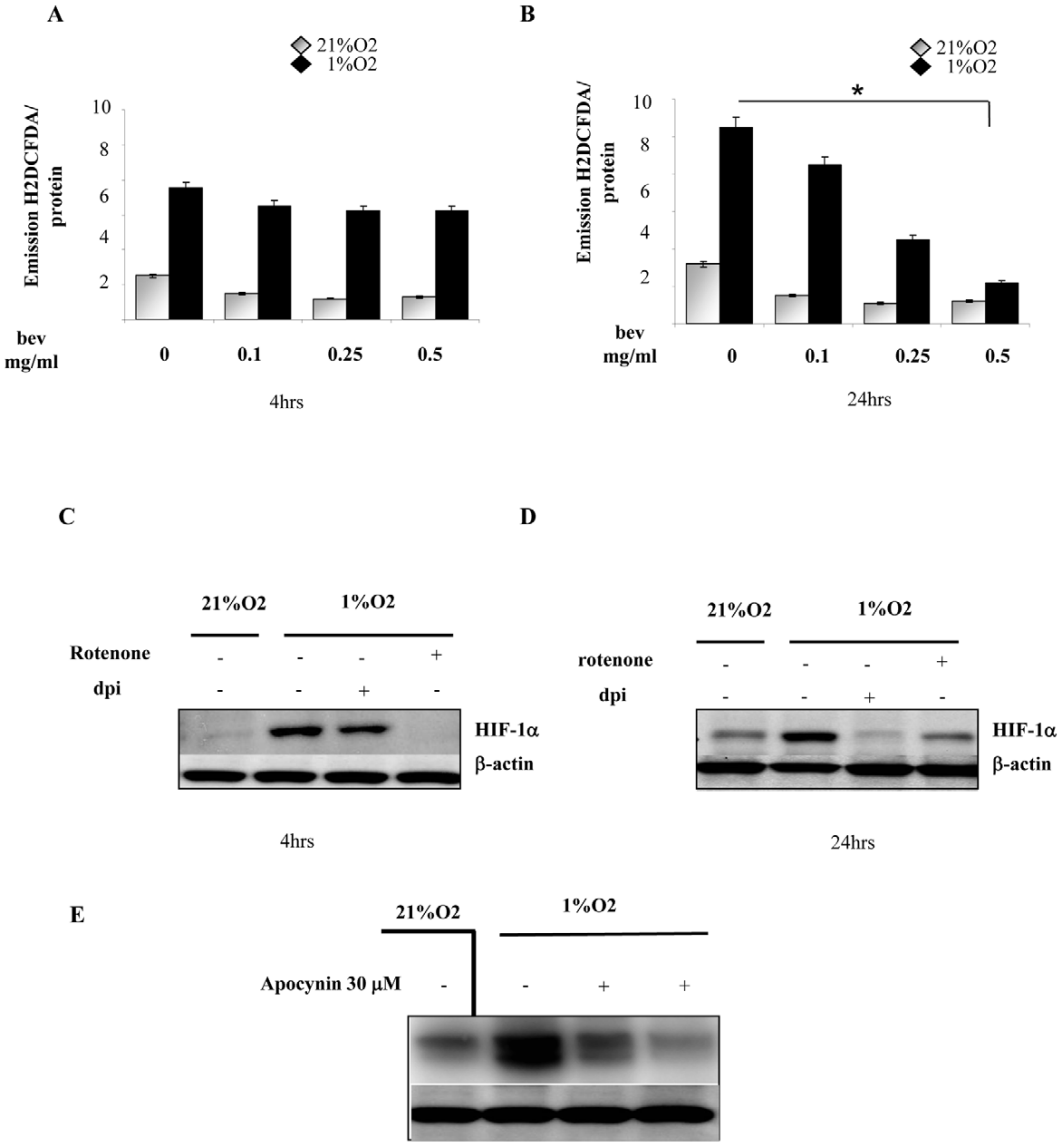
Bevacizumab inhibits ROS production under hypoxia. (**A, B**) Hs29-4T cells were serum-starved for 24 h. Cells were then incubated under normoxia or hypoxia of increasing concentrations of bevacizumab (*Bev*) for 4 and 24 h. Hydrogen peroxide production was evaluated with DCDF-DA and normalized on protein content. *P<0,001 versus untreated control under hypoxia. (**C, D**) HIF-1α expression was assessed by Western blotting in Hs29-4T cells serum-starved for 4 and 24 h and incubated under normoxia or hypoxia in the absence or presence of DPI (5 µM) and rotenone (1 µM) for additional 24 h. (**E**) HIF-1α expression was assessed by Western blotting in Hs29-4T cells serum-starved for 24 h and incubated under normoxia or hypoxia in the presence of Apocynin (30 µM) for additional 4 and 24 h.

ROS act as intracellular messengers following NADPH oxidase activation due to membrane receptors activation, by a variety of bioactive peptides including growth factors, cytokines and hormones. Therefore we suppose that, under hypoxia, VEGF-A stimulates NADPH oxidase to produce a second wave of ROS, which are able to further sustain HIF-1α stabilization. Exposure of melanoma cells to VEGF at 5, 10, 15, and 30 min leads to a significant rise of intracellular ROS both in normoxia and hypoxia peaking at 15 minutes after stimulation. This effect is strongly amplified under hypoxic conditions (Fig. 6A). To validate if autocrine VEGF-A affects HIF-1α protein stabilization during hypoxia, Hs29-4T cells were treated with the neutralizing antibody for VEGF-A (Fig. 6B), or with Bevacizumab under normoxic and hypoxic conditions for 4 and 24 h (Fig. 6C). Both VEGF-A neutralizing antibody and Bevacizumab, abrogate hypoxic induction of HIF-1α only at 24 h. To identify the specific receptor used by autocrine VEGF-A in hypoxia, Hs29-4T cells were treated with VEGF-R1 and VEGF-R2 neutralizing antibodies for 24 h. The results indicate that VEGF-R2 neutralizing antibody inhibits HIF-1α protein accumulation, suggesting VEGF-R2 as the key mediator of hypoxia-induced survival (Fig. 6D). To analyse the differential time course of early and late ROS during hypoxia exposure of melanoma, we treated we treated Hs29-4T with Bevacizumab for 4 and 24 h. The obtained data show that Bevacizumab is able to inhibit ROS delivery under hypoxic conditions, in a dose dependent fashion, only after 24 h (Fig. 7A, B). Moreover we observed that early inhibition of NADPH oxidase by DPI does not affect HIF-1α, whereas rotenone efficiently blocks HIF-1α accumulation (Fig. 7C, D). These observation is further confirmed by stimulation of Hs29-4T cells with Apocynin, used again at 4 and 24 hrs. In keeping, this selective NADPH inhibitor is more efficient in inhibiting HIF-1α protein accumulation at 24 hrs, than earlier.

**Figure 8:**
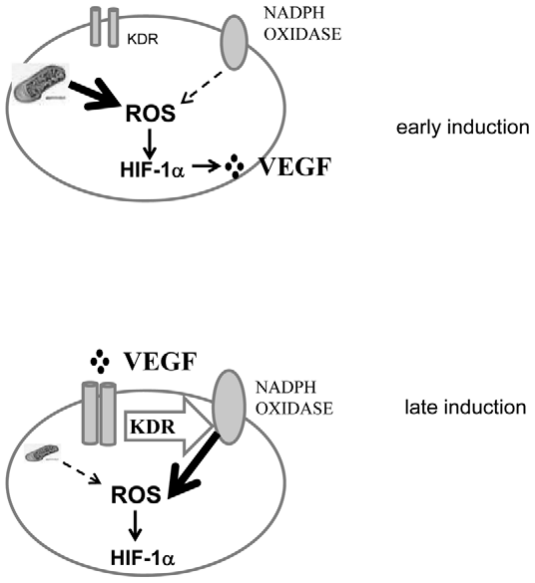
Schematic illustration of VEGF autocrine loop in HS-294T. Early induction of HIF-1α mediated by mitochondrial ROS and late induction of HIF-1α mediated by NADPH oxidase.

Therefore, it is likely that hypoxia engages two different ROS sources, being mitochondria the earliest one and NADPH oxidase the latest. We propose that mitochondrial ROS delivery behaves as the start button, when hypoxic exposure begins, but later on, VEGF-A secreted owing to the early HIF-1 signalling, through its receptor VEGF-R2, elicits a NADPH oxidase-driven second wave of ROS, able to further sustain HIF-1α expression leading to cells survival (Fig. 8).

## Discussion

Metastatic melanoma is a very aggressive tumour, unfortunately very resistant to chemotherapy. Etoposide and cisplatin-based combination therapy has only partial effects for patients survival, limiting to 10% the effective responsiveness [25]. The exact mechanisms exploited by aggressive melanoma to achieve this high intrinsic drug resistance is currently unknown and is at present a real challenge for molecular oncologists. Indeed, up to now immunotherapy using interleukin-2 and interferon-γ is the most used pharmacological approach for this kind of neoplasia [26]. Herein we report that etoposide resistance in human aggressive melanoma Hs29-4T cells is tightly correlated to a two waved redox-based VEGF autocrine loop, exploiting hypoxic environment. Our data indicate that: i) 1% O_2_ hypoxia protects cells from etoposide-induced cell death through an early ROS delivery by mitochondria; ii) this leads to a redox stabilization of HIF-1 leading to autocrine secretion of VEGF-A; iii) VEGF-A acts as a survival factor for melanoma cells through interaction with its receptor VEGFR2, leading to generation of a second wave of ROS through NADPH oxidase, which ultimately causes a long lasting HIF-1 stabilization, enhancing melanoma cell survival to etoposide chemotherapy.

Etoposide is known, as many other chemotherapic agents, to cause apoptosis by increasing ROS, leading cells to overwhelming toxic threshold. Of note, in our experimental model etoposide did not increase ROS to toxic concentration, even in normoxia, likely due to the high intrinsic antioxidant/scavenger ability of melanoma cells [27], [28]. In keeping with this, melanoma cells are highly sensitive to changes in the intracellular redox balance and actively respond exploiting these extra ROS by engaging motility [17] and, as demonstrated herein, chemoresistance. We also excluded a direct effect of etoposide, as a known topoisomerase II inhibitor, on HIF-1 protein stabilization. Our data indicate HIF-1 as the driver of hypoxic-dependent pathway mediating melanoma survival and in particular we emphasize the role of its redox-based stabilization due to ROS delivery during hypoxia. This conclusion was supported by experiments performed using siRNA targeting HIF-1α, which completely blocked hypoxia-induced resistance to etoposide. HIF-1 has already been proposed as a key player of cancer cells survival, and mechanisms proposed are mainly correlated to its ability to prompt anaerobic glycolitic metabolism, or expression of anti-apoptotic proteins, as Bcl-2, Bcl-XL or down regulation of pro-apoptotic proteins such as Bid or Bax [29], [30]. In melanoma cells HIF-1 drives a survival response mainly mediated by the autocrine production of VEGF-A, acting on melanoma cells themselves and eliciting canonical Akt-dependent pro-survival signals.

Intratumoural hypoxia can produce several different effects on cancer cells, ranging from metabolic reprogramming towards a glycolitic phenotype, over-expression of ABC transporters, selection of mutated cells whose apoptotic process is deficient, or protection from apoptotic inducers. Indeed, hypoxic cancer cells are more invasive, resistant to apoptosis and ultimately to chemotherapy and radiation [31] therapy [8], [32]. Moreover mounting evidence indicate that hypoxic cancer cells undergo exposure to oxidative stress, thereby developing adaptive strategies to survive to the hostile milieu [33]. Of note hypoxic cells can enhance their antioxidant capacity and hypoxia can behave as a promoting factor for this behaviour, with a possible correlation with resistance to therapy [5], [34]. We also recently reported that aggressive melanoma cells respond to hypoxia engaging a motogen escaping strategy, based on redox stabilization of HIF-1 and activation of the Met protoncogene, allowing a proteolytic motility enhancing metastatic dissemination to lungs [17].

Our data contribute to the long-lasting debate about the role of ROS in oxygen sensing and activation of HIF-1α [12], [28], [35], [36]. Under normoxia, a variety of stimuli including growth factors, hormones, vasoactive peptides, metal ions, hydrogen peroxide, and certain nitric oxide donors, are known to stabilize HIF-1α, using at least in part redox-based mechanisms. During mild hypoxia (1–3% O_2_), at least two mechanisms have been proposed to explain the paradoxical production of ROS during oxygen lack. First, mitochondrial ROS have been involved, through deregulation of complex III of electron transfer chain at the level of Fe-S Rieske protein. Second, while mitochondrial ROS appear a main font during hypoxia, some recent studies also support the activation of Rac-1/NADPH oxidase complex through Akt or Src-mediated pathways [20], [37]. Whatever the mechanism to produce ROS, increased generation of OH^·^ from H_2_O_2_ through a Fenton reaction, is acknowledged to promote the conversion of Fe^2+^ to Fe^3+^, leading to PHD inactivation. This ROS-mediated inhibition of PHDs, enzymes devoted to oxygen-dependent degradation of HIF-1, has been involved in the redox-dependent stabilization of HIF-1. Another proposed mechanism of oxidative stabilization of HIF-1α is that ROS activate multiple signalling pathways, such as Akt and p38MAPK, which may render PHD catalytically inactive [20], [38].Of note, attenuation of ROS levels by antioxidants (such as N-acetyl cysteine, ascorbate, and catalase, or genetic down-modulation of NADPH oxidase-4, were found to decrease HIF-1α expression [21], [39]. In keeping with the key role exerted by ROS in sensing the effects of hypoxia, Gao et al reported that the anti-tumourigenic effect of antioxidants as N-acetyl cysteine and vitamin C in murine models of Myc-mediated tumourigenesis are indeed HIF-1-dependent [15]. We herein confirm for hypoxic ROS a key role for HIF-1 stabilization and activation of its transcriptional response, but we identify two distinct waves of ROS delivery. The first one is mediated by mitochondria, as indicated by rotenone treatment, which decreases the electron flux from complex I and hence inhibits ROS delivery from the downstream complex III, or by silencing the Rieske protein, which is the direct responsible for ROS delivery. The second wave is driven by NADPH oxidase activation in response to VEGF-A interaction with its VEGF-R2. Of note the first wave of mitochondrial ROS plays a mandatory role in eliciting the autocrine production of VEGF-A from melanoma cells, as revealed by the effect of its block on downstream events as VEGF-A expression or VEGF-R2 activation. This second wave of NADPH-driven ROS concurs to a further redox-based stabilization of HIF-1. Although the involvement of early and late ROS in cancer cells survival to chemotherapeutic agents is a novel finding, VEGF-A has already been involved in an autocrine survival loop in hypoxic colon cancer cells [40]. In keeping with the key role exerted by VEGF-A for chemoresistance, bevacizumab, a humanized anti-VEGF monoclonal antibody increases the efficacy of chemotherapy for the treatment of metastatic colorectal cancer [41], [42]. Here we report that bevacizumab is also active in aggressive melanoma cells, as it completely reverts the hypoxic induced survival, increasing sensitivity to etoposide. In keeping with our model (Fig. 8), bevacizumab is active in inhibiting the late, VEGF-dependent, HIF-1 activation. On the basis of these results we propose that in aggressive melanoma, the presence of a functional VEGF-A/VEGF-R2 autocrine loop, should render cells more resistant to chemotherapy-induced apoptosis under hypoxic stressing condition, which may contribute to treatment failure. Expression of VEGF-R(s) has been correlated with stem-like traits and CD133 expression, and hypoxia is acknowledged to be a favourable environmental condition for the maintenance of the stem cell phenotype [43], [44]. We can therefore speculate that in melanoma hypoxia could select VEGF-A sensitive/VEGF-R2 positive populations, in which the two waves of ROS synergize to grant survival and resistance to chemotherapy. In keeping with this idea, recently it has been reported that in skin cancers blocking VEGFR2 caused tumour regression by impairing cancer stem cells renewal properties, suggesting that, besides its well-known effect on angiogenesis, VEGF affects tumour growth by promoting cancer stem cells expansion [45]. Therefore, beside its acknowledged role for hematopoietic malignancies, VEGF-R2 should be proposed as a stem-like marker also in solid tumors. Future study should explore the possibility that VEGFR2-positive cells represent the more malignant, therapy resistant, tumor cells. Hence, our data propose the couple HIF-1/VEGF-A as mandatory players in melanoma resistance to therapy, acting as a reciprocal redox based circuitry. Future studies combining anti-VEGF and antioxidant therapies should therefore have important implications for treatment of malignant melanoma.

## Materials and Methods

### Materials

Unless specified all reagents were obtained from Sigma. Hs29-4T cells were from ATCC, all antibodies were from Santa Cruz Biotechnology, except the anti-HIF-1α which was from BD. DCF-DA was from Molecular Probes. PVDF was from Millipore. siRNA oligonucleotides targeting HIF-1α (target sequence: 5′-AAAGGACAAGUCACCACAGGA-3′) and siRNA targeting the Rieske iron-sulfur protein (target sequence 5′-AAUGCCGUCACCCAGUUCGUU-3′) were obtained from Qiagen. Annexin-V fluorescein isothiocyanate apoptosis kit was from Roche Diagnostic. Caspase Assay kit was from Immunoistochemistry technologies.

### Cell culture and transfections

Hs29-4T cells were cultured in DMEM supplemented with 10% FCS, in 10% CO_2_ humidified atmosphere. Experiments under hypoxic condition (1% O_2_) were performed in the hypoxic incubator. For transient transfections, Hs29-4T cells were plated in 60-mm cell culture dishes and grown to 80% confluence. The siRNA was diluted to a final concentration of 20 nM. Transfections were performed using Lipofectamine (Invitrogen), following manufacturer's recommendations 48 h before treatment with the indicated conditions.

### Flow cytometry and apoptotic cells analysis

Annexin-V staining was performed using the Annexin-V fluorescein isothiocyanate apoptosis kit according to the manufacturer's instructions. Gated cells were plotted on a dot-plot showing Annexin-V staining and propidium iodide (PI) staining. Index of apoptotic cells was determinate summing Annexin V positive (early apoptotic) cells and Annexin/PI positive (late apoptotic).

### Western blot analysis

Hs29-4T (1×10^6^) cells derived from our experimental conditions were lysed for 20 min on ice in 500 µl of complete RIPA lysis buffer (50 mM Tris-HCl, pH 7.5, 150 mM NaCl, 1% Nonidet P-40, 2 mM EGTA, 1 mM sodium orthovanadate, 1 mM phenyl-methanesulphonyl-fluoride, 10 µg/ml aprotinin, 10 µg/ml leupeptin). Lysates were separated by SDS/PAGE, and transferred onto nitrocellulose. Immunoblots were incubated in 3% BSA, 10 mM Tris/HCl (pH 7.5), 1 mM EDTA, and 0.1% Tween 20, for 1 h at room temperature, probed first with specific antibodies and then with secondary antibodies.

### Tetramethylrhodamine-methyl ester (TMRM) staining and imaging

For mitochondria visualization in living cells, Hs29-4T cells treated as previously described were stained for 15 min at 37°C with 1 µM TMRM. Loading of TMRM in metabolically active mitochondria is driven by mitochondrial membrane potential that is well maintained by healthy living cells. The disruption of this membrane potential causes an abrupt decrease in mitochondrial fluorescence that is a distinctive feature of programmed cell death. This potentiometric dye (excitation 543 nm, emission 590 nm) was quantified by fluorimetric assay (FluoroSkan).

### Assay of intracellular ROS

To evaluate the intracellular production of H_2_O_2_, DCF-DA was added (final concentration, 5 µM) 3 min before the end of the incubation time. Cells were lysed in 1 ml of RIPA buffer and analysed immediately by fluorescence analysis using a Perkin Elmer Fluorescence Spectrophotometer 650-10S equipped with a Xenon Power Supply (excitation 488 nm, emission 510 nm).

### Statistical analysis

Data are presented as means ± SD from at least three independent experiments. Statistical analysis of the data was performed using the Student t test. P values of ≤0.001were considered statistically significant.
